# Supplementary figures and images for: Analysis of the p53 pathway in peripheral blood of retinoblastoma patients; potential biomarkers
Source: PLoS One. 2020 Jun 5;15(6):e0234337. doi: 10.1371/journal.pone.0234337 (PMC7274427; doi:10.1371/journal.pone.0234337)

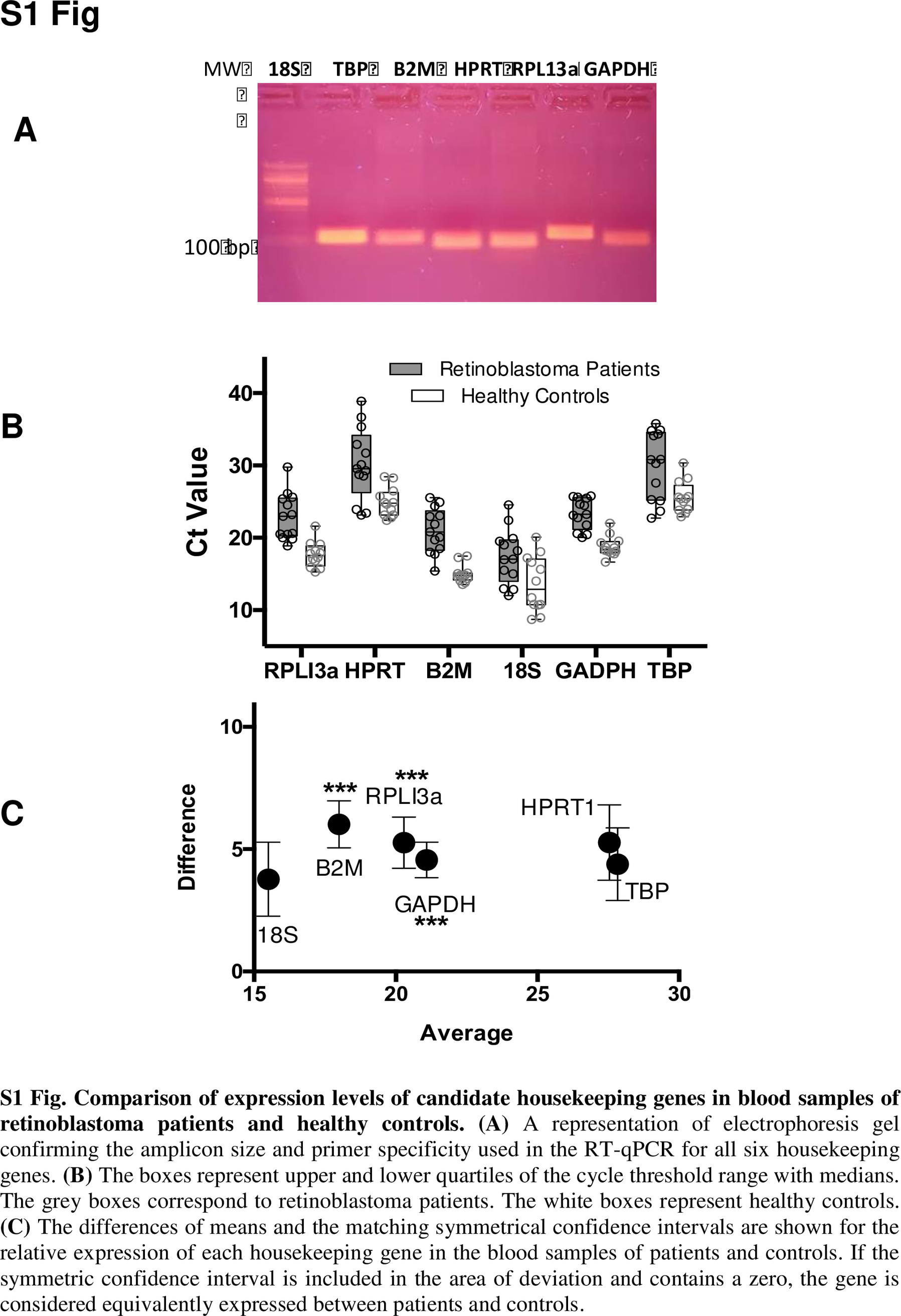

Supplement: S1 Fig — (A) A representation of electrophoresis gel confirming the amplicon size and primer specificity used in the RT-qPCR for all six housekeeping genes. (B) The boxes represent upper and lower quartiles of the cycle threshold range with medians. The grey boxes correspond to retinoblastoma patients. The white boxes represent healthy controls. (C) The differences of means and the matching symmetrical confidence intervals are shown for the relative expression of each housekeeping gene in the blood samples of patients and controls. If the symmetric confidence interval is included in the area of deviation and contains a zero, the gene is considered equivalently expressed between patients and controls. (TIF) [file pone.0234337.s001.tif]
