# Supplementary material for: Analysis of the p53 pathway in peripheral blood of retinoblastoma patients; potential biomarkers
Source: PLoS One. 2020 Jun 5;15(6):e0234337. doi: 10.1371/journal.pone.0234337 (PMC7274427; doi:10.1371/journal.pone.0234337)
Supplement: S1 Table — (DOC) [file pone.0234337.s002.doc]

**S1 Table. Housekeeping genes selected for expression analysis.**

| **Gene name** | **Abbreviation** | **Genebank access No** | **Function** |
| --- | --- | --- | --- |
| **Glyceraldehyde-3-phopsphate dehydrogenase** | GAPDH | NM_002046 | Enzyme that catalyzes the sixth step of glycolytic pathway |
| **Hypoxanthine phosphoribosyltransferase 1** | HPRT1 | NM_000194 | Enzyme working in the generation of purines nucleotides through the purine salvage pathway |
| **β-2-microglobulin** | B2M | NM_004048 | Is a component of the class 1 major histocompatibility complex (MHC) |
| **TATA-binding protein** | TBP | NM_003194 | Transcription factor |
| **Ribosomal protein L13a** | RPL13a | NM_012423 | Structural component of the large 60S ribosomal subunit |
| **18S Ribosomal RNA** | 18S | NR_003286 | Structural component of the small 40S ribosomal subunit |
