# Supplementary material for: Analysis of the p53 pathway in peripheral blood of retinoblastoma patients; potential biomarkers
Source: PLoS One. 2020 Jun 5;15(6):e0234337. doi: 10.1371/journal.pone.0234337 (PMC7274427; doi:10.1371/journal.pone.0234337)
Supplement: S2 Table — (DOC) [file pone.0234337.s003.doc]

**S2 Table. Statistical analysis of housekeeping level**

| **Gene** | **Mann-Whitney-Wilcoxon** | **CV patients** | **CV controls** | **CV total** |
| --- | --- | --- | --- | --- |
| **GAPDH** | 0.0014 ^ | 9.73 | 7.81 | 14.67 |
| **HPRT** | 0.0532 | 14.13 | 8.16 | 13.42 |
| **B2M** | 0.0003 ^ | 12.27 | 8.48 | 19,17 |
| **TBP** | 0.2670 | 18.14 | 8.88 | 14.47 |
| **RPL13a** | 0.0011 ^ | 15.64 | 10.31 | 18.85 |
| **18S** | 0.0750 | 20.28 | 28.21 | 26.29 |

Non-parametric Wilcoxon-Mann-Whitney test, highest values indicate best candidates ^ <0.05 indicates unsuitable housekeeping genes

**S3 Table. Stability ranking of the housekeeping genes**

| **Gene** | **Norm Finder** | **Ranking** | **RefFinder** | **Ranking** | **GeNorm** | **Ranking** |
| --- | --- | --- | --- | --- | --- | --- |
| **HPRT** | 0.016 | 1 | 1.32 | 1 | 1.45 | 1-2 |
| **TBP** | 0.024 | 2 | 1.68 | 2 | 1.45 | 1-2 |
| **18S** | 0.097 | 3 | 2.28 | 3 | 4.13 | 3 |
| **Best combination two genes** | 0.017 HPRT/TBP | |  |  |  |  |
