# Supplementary material for: Analysis of the p53 pathway in peripheral blood of retinoblastoma patients; potential biomarkers
Source: PLoS One. 2020 Jun 5;15(6):e0234337. doi: 10.1371/journal.pone.0234337 (PMC7274427; doi:10.1371/journal.pone.0234337)
Supplement: S3 Table — (DOC) [file pone.0234337.s004.doc]

**S3 Table. Stability ranking of the housekeeping genes**

| **Gene** | **Norm Finder** | **Ranking** | **RefFinder** | **Ranking** | **GeNorm** | **Ranking** |
| --- | --- | --- | --- | --- | --- | --- |
| **HPRT** | 0.016 | 1 | 1.32 | 1 | 1.45 | 1-2 |
| **TBP** | 0.024 | 2 | 1.68 | 2 | 1.45 | 1-2 |
| **18S** | 0.097 | 3 | 2.28 | 3 | 4.13 | 3 |
| **Best combination two genes** | 0.017 HPRT/TBP | |  |  |  |  |
