# Supplementary material for: Analysis of the p53 pathway in peripheral blood of retinoblastoma patients; potential biomarkers
Source: PLoS One. 2020 Jun 5;15(6):e0234337. doi: 10.1371/journal.pone.0234337 (PMC7274427; doi:10.1371/journal.pone.0234337)
Supplement: Attachment — Submitted filename: Comments to the Author.docx [file pone.0234337.s005.docx]

PONE-D-20-08362
Analysis of the p53 pathway in peripheral blood of retinoblastoma patients; potential biomarkers
PLOS ONE

Dear Dra Olivares-Ilana,

Thank you for submitting your manuscript to PLOS ONE. After careful consideration, we feel that it has merit but does not fully meet PLOS ONE’s publication criteria as it currently stands. Therefore, we invite you to submit a revised version of the manuscript that addresses the points raised during the review process.

We would appreciate receiving your revised manuscript by May 31 2020 11:59PM. When you are ready to submit your revision, log on to [https://www.editorialmanager.com/pone/](https://www.editorialmanager.com/pone/) and select the 'Submissions Needing Revision' folder to locate your manuscript file.

If you would like to make changes to your financial disclosure, please include your updated statement in your cover letter.

____________________________________________________________________________________________________________________________

Dear Sumitra Deb,

Thank you very much for your kind letter and the reviewer´s response. We are very happy to read your letter and the comments have been very useful. A proofreading PRS service has revised the MS, we add the certificate from the company as a supplementary information.

We would like to update our funding statement

Please find our response to each comment below in italic.

Journal requirements:

When submitting your revision, we need you to address these additional requirements:

1.    Please ensure that your manuscript meets PLOS ONE's style requirements, including those for file naming. The PLOS ONE style templates can be found at [http://www.plosone.org/attachments/PLOSOne_formatting_sample_main_body.pdf](http://www.plosone.org/attachments/PLOSOne_formatting_sample_main_body.pdf) and [http://www.plosone.org/attachments/PLOSOne_formatting_sample_title_authors_affiliations.pdf](http://www.plosone.org/attachments/PLOSOne_formatting_sample_title_authors_affiliations.pdf)

*Done*

2. Please provide additional details regarding participant consent. We note that you state "Written/signed informed consent was obtained from the guardians on behalf of the minors involved in the study". Please also state in the Methods and online submission information whether informed consent was obtained from all subjects in this study and whether it was written or verbal. If the need for consent was waived by the ethics committee, please include this information. For additional information about PLOS ONE ethical requirements for human subjects research, please refer to [http://journals.plos.org/plosone/s/submission-guidelines#loc-human-subjects-research](http://journals.plos.org/plosone/s/submission-guidelines#loc-human-subjects-research).

*Done, see page 5 on the MS*

3. Please add the sources and catalog numbers of your reagents as well as details regarding all equipment used in your study to the Methods section of your manuscript.

*Done*

4. We suggest you thoroughly copyedit your manuscript for language usage, spelling, and grammar. If you do not know anyone who can help you do this, you may wish to consider employing a professional scientific editing service.  

Whilst you may use any professional scientific editing service of your choice, PLOS has partnered with both American Journal Experts (AJE) and Editage to provide discounted services to PLOS authors. Both organizations have experience helping authors meet PLOS guidelines and can provide language editing, translation, manuscript formatting, and figure formatting to ensure your manuscript meets our submission guidelines. To take advantage of our partnership with AJE, visit the AJE website (http://learn.aje.com/plos/) for a 15% discount off AJE services. To take advantage of our partnership with Editage, visit the Editage website ([www.editage.com](http://www.editage.com/)) and enter referral code PLOSEDIT for a 15% discount off Editage services.  If the PLOS editorial team finds any language issues in text that either AJE or Editage has edited, the service provider will re-edit the text for free.

Upon resubmission, please provide the following:

●      The name of the colleague or the details of the professional service that edited your manuscript

●      A copy of your manuscript showing your changes by either highlighting them or using track changes (uploaded as a *supporting information* file)

●      A clean copy of the edited manuscript (uploaded as the new *manuscript* file)

5. PLOS ONE now requires that authors provide the original uncropped and unadjusted images underlying all blot or gel results reported in a submission’s figures or Supporting Information files. This policy and the journal’s other requirements for blot/gel reporting and figure preparation are described in detail at [https://journals.plos.org/plosone/s/figures#loc-blot-and-gel-reporting-requirements](https://journals.plos.org/plosone/s/figures#loc-blot-and-gel-reporting-requirements) and [https://journals.plos.org/plosone/s/figures#loc-preparing-figures-from-image-files](https://journals.plos.org/plosone/s/figures#loc-preparing-figures-from-image-files). When you submit your revised manuscript, please ensure that your figures adhere fully to these guidelines and provide the original underlying images for all blot or gel data reported in your submission. See the following link for instructions on providing the original image data: [https://journals.plos.org/plosone/s/figures#loc-original-images-for-blots-and-gels](https://journals.plos.org/plosone/s/figures#loc-original-images-for-blots-and-gels).

In your cover letter, please note whether your blot/gel image data are in Supporting Information or posted at a public data repository, provide the repository URL if relevant, and provide specific details as to which raw blot/gel images, if any, are not available. Email us at [plosone@plos.org](mailto:plosone@plos.org) if you have any questions.

*We add the uncropped and unadjusted gel in S1 Figure.*

6. Thank you for stating the following in the Acknowledgments Section of your manuscript:

"This work was supported by Conacyt CB-256637. This work, including the efforts of
Vanesa Olivares-Illana, was funded by L´oreal-UNESCO-AMC. JH-M Cátedras
CONACyT program. We thanks to all patients and families."

We note that you have provided funding information that is not currently declared in your Funding Statement. However, funding information should not appear in the Acknowledgments section or other areas of your manuscript. We will only publish funding information present in the Funding Statement section of the online submission form.

Please remove any funding-related text from the manuscript and let us know how you would like to update your Funding Statement. Currently, your Funding Statement reads as follows:

"The funders had no role in study design, data collection and analysis, decision to publish, or preparation of the manuscript."

*Yes please, we would like to update our funding statement*

7. Please include captions for your Supporting Information files at the end of your manuscript, and update any in-text citations to match accordingly. Please see our Supporting Information guidelines for more information: <http://journals.plos.org/plosone/s/supporting-information>.

*Done*

**Comments to the Author**

1. Is the manuscript technically sound, and do the data support the conclusions?

The manuscript must describe a technically sound piece of scientific research with data that supports the conclusions. Experiments must have been conducted rigorously, with appropriate controls, replication, and sample sizes. The conclusions must be drawn appropriately based on the data presented.

Reviewer #1: Partly

Reviewer #2: Partly

2. Has the statistical analysis been performed appropriately and rigorously?

Reviewer #1: Yes

Reviewer #2: Yes

3. Have the authors made all data underlying the findings in their manuscript fully available?

The [PLOS Data policy](http://www.plosone.org/static/policies.action#sharing) requires authors to make all data underlying the findings described in their manuscript fully available without restriction, with rare exception (please refer to the Data Availability Statement in the manuscript PDF file). The data should be provided as part of the manuscript or its supporting information, or deposited to a public repository. For example, in addition to summary statistics, the data points behind means, medians and variance measures should be available. If there are restrictions on publicly sharing data—e.g. participant privacy or use of data from a third party—those must be specified.

Reviewer #1: Yes

Reviewer #2: No *(Now we add the full size gel in S1 Figure)*

4. Is the manuscript presented in an intelligible fashion and written in standard English?

PLOS ONE does not copyedit accepted manuscripts, so the language in submitted articles must be clear, correct, and unambiguous. Any typographical or grammatical errors should be corrected at revision, so please note any specific errors here.

Reviewer #1: No *(the MS was send to proofreading service)*

Reviewer #2: Yes

5. Review Comments to the Author

Please use the space provided to explain your answers to the questions above. You may also include additional comments for the author, including concerns about dual publication, research ethics, or publication ethics. (Please upload your review as an attachment if it exceeds 20,000 characters)

Reviewer #1: In this manuscript, the authors have attempted to monitor the status of the p53 pathway in peripheral blood samples obtained from retinoblastoma patients. They measure the mRNA levels of p53, RB, MDMX and MDM2 which could be further used as biomarkers for early diagnosis. Although it is an interesting premise and could be a good diagnostic approach, I am not able to understand the clear rationale behind utilizing the blood samples of RB patients to analyze the p53 pathway. The authors are requested to elaborate more on this.

*It has been shown that the p53 pathway is, indeed altered in retinoblastoma tumours, if this could be one of the causes of the diseases as has been proposed for several authors, then we thought that it could have a hereditary component, meaning that could be studied in any cell of the body. That was also the motivation to study the family members. We add more about it in page 4 and page 14 on the MS.*

Moreover, other signaling pathways like the Wnt signaling is also highly functional in RB and could have higher significance as compared to p53. Have the authors looked into these pathways (mRNA analysis of beta-catenin etc)?

*The reviewer is right. Other pathways indeed could also be potential biomarkers for retinoblastoma. Has recently been published a review paper about it:*

*Jie Sun , Hui-Yu Xi1,2, Qing Shao and Qing-Huai Liu. Biomarkers in retinoblastoma. Int J Ophthalmol, Vol. 13, No. 2, Feb.18, 2020.*

*In the paper, the authors revised the potential biomarkers for retinoblastoma and both Wnt and p53 pathways, among many others, are mentioned. In this particular work, we focus on p53.*

Depending on the severity of RB, as mentioned, it could be unilateral or bilateral (simultaneously in both eyes) or in cases, it could start off as unilateral and at a later stage it could become bilateral. How could the presented approach address this issue?

*This is an interesting question that we have been thinking about. We thought that a hereditary component of the p53 pathways is present. Then we could imagine that the bilateral cases would present higher significant differences in the levels of p53, MDMX or MDM2, more than the unilateral. However, this is difficult to demonstrate since, as the reviewer explains, a patient that at the moment of the study is unilateral, can later become bilateral. However, we found a correlation between the age of diagnosis and the levels of expression of p53 and MDMX m mRNA, and as has been postulated that the age of diagnosis in correlated with the laterality.*

Also, peripheral blood would have circulating tumor DNA that could be coming from tumors that have metastasized from the retina to other parts of the body? In that case, would the diagnosis mentioned here delineate between the original vs metastasized tumor that could have a totally different p53 signature? The authors are recommended to discuss these in detail.

*Of course, this is true, and it was our omission because we did not explain the inclusion/exclusion criteria in the MS. Now we add the following information on page 5 of the MS:*

*Inclusion criteria: Children with the diagnosis of retinoblastoma, without a known diagnosis of other cancer. Exclusion criteria: Patients with a diagnosis of other types of cancer. Controls: Healthy children at pediatric age and healthy adults in the same range of age of the family member patients.*

*On the other hand non of the participating patient's in the study developed metastasis. Now we add a table with the characteristics of the patients in the MS.*

Optional: This may or may not be addressed. What about the more rarer cases of trilateral RB? Have the authors looked into the p53 signaling in these cases? If not, it would be good to include a few points about the same if possible.

*This is a good suggestion; unfortunately we were unable to obtain the sample of a patient with these characteristics.*

Minor Changes:
1. Introduction...Line 1...Change "Paediatric" to "Pediatric".

*Done*

2. The grammar needs to be formatted. There are lots of places where the sentences need corrections e.g. "it has been used to search of early biomarkers (Introduction)...should be changed to "it has been used to search or identify early biomarkers".

*Thank you, the correction is done and the MS has been carefully formatted*

3. Proteasome 26 S needs to be written without any space.

*Done*

4. I do not see the significance of writing about the housekeeping genes in detail. It could be just mentioned in a sentence or two.

We reduced this part, and sent the Figure 1 (about the housekeeping genes) to Supplemental information.

5. The authors are requested to include these references:
a.[https://www.ncbi.nlm.nih.gov/pubmed/16231976](https://www.ncbi.nlm.nih.gov/pubmed/16231976)
b.[https://www.ncbi.nlm.nih.gov/pubmed/15190215](https://www.ncbi.nlm.nih.gov/pubmed/15190215)
*Thank you for the suggestions, we added them!*

Reviewer #2: In this manuscript authors have analyzed peripheral blood samples from patients suffering from retinoblastoma for mRNA levels of RB, p53, MDM2, MDMX and investigated the involvement of these genes in the development of cancer. There were some minor issues with the manuscript grammar and use of appropriate scientific language.

*Done*

The authors need to provide complete profiling of the RB and p53 genes in the samples through sequencing and the status of mutations in them.

*It is a good idea to get the mutation spectrum of RB and p53 in the patients. However, this is not the focus of the present work. In this manuscript we focus on the analysis of the levels of mRNA of the p53 pathways genes and RB. We will consider studying the mutation status of these genes for a future work.*

The authors also show the comparison in the gene levels between patients, controls and healthy family in the same graph.
While the analysis shows some significant correlations between the p53, MDM2 and RB mRNA levels in patients and controls, some of the associations are not novel like that between p53 and MDM2.

*The reviewer is right, the correlation between p53 and MDM2 it has been very well documented. Our results corroborates that in blood samples of retinoblastoma patients the correlation do not changes due to the disease.*

The sample size and the scope of analysis performed in this manuscript is not sufficient enough at this point to prove the use of these gene signatures as potential biomarkers.

*We agreed; however, we hope that our work will stimulate others too and together, we can finally found a genes signature for this childhood cancer.*
